# Supplementary figures and images for: Assessing nanobody interaction with SARS-CoV-2 Nsp9
Source: PLoS One. 2024 May 17;19(5):e0303839. doi: 10.1371/journal.pone.0303839 (PMC11101046; doi:10.1371/journal.pone.0303839)

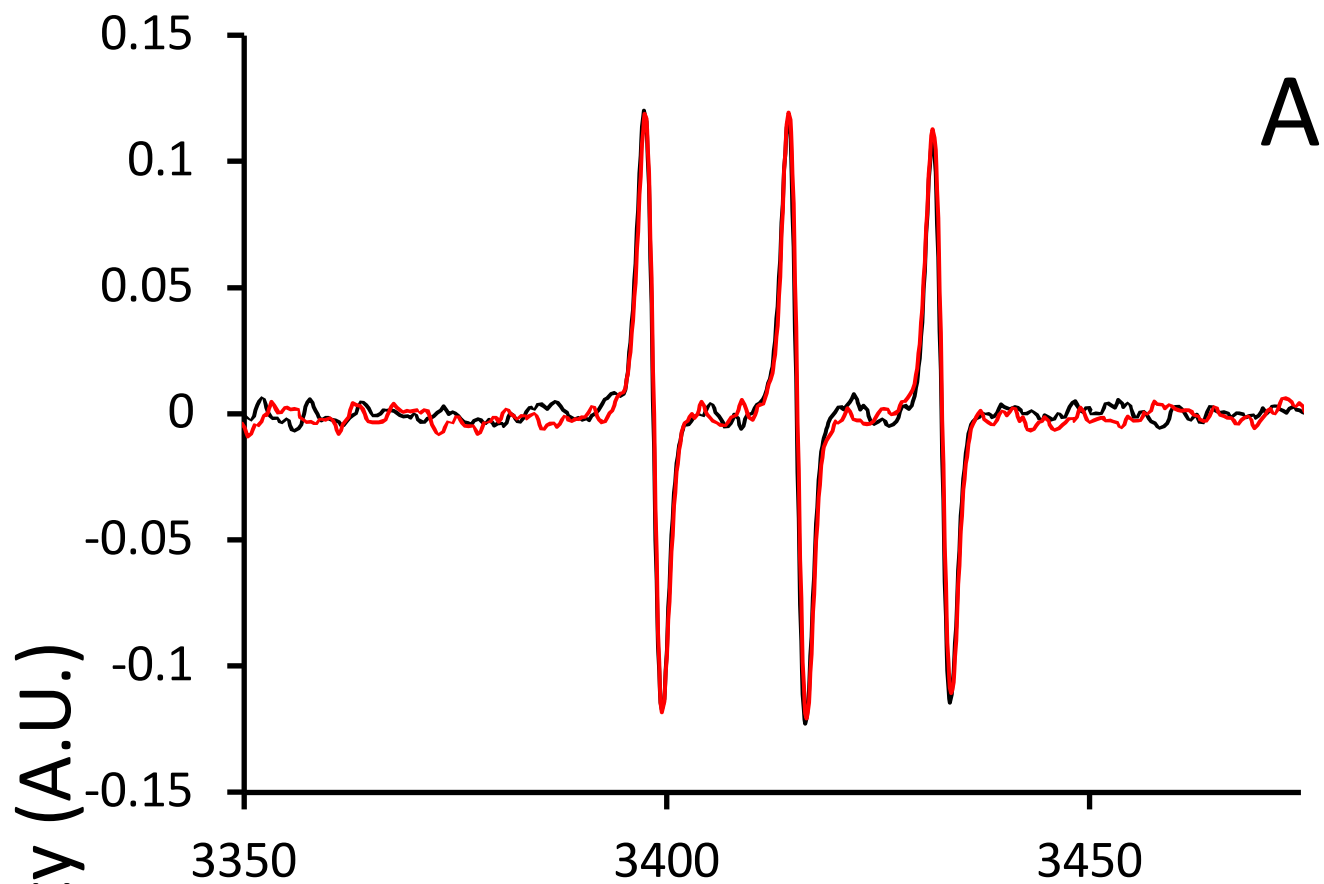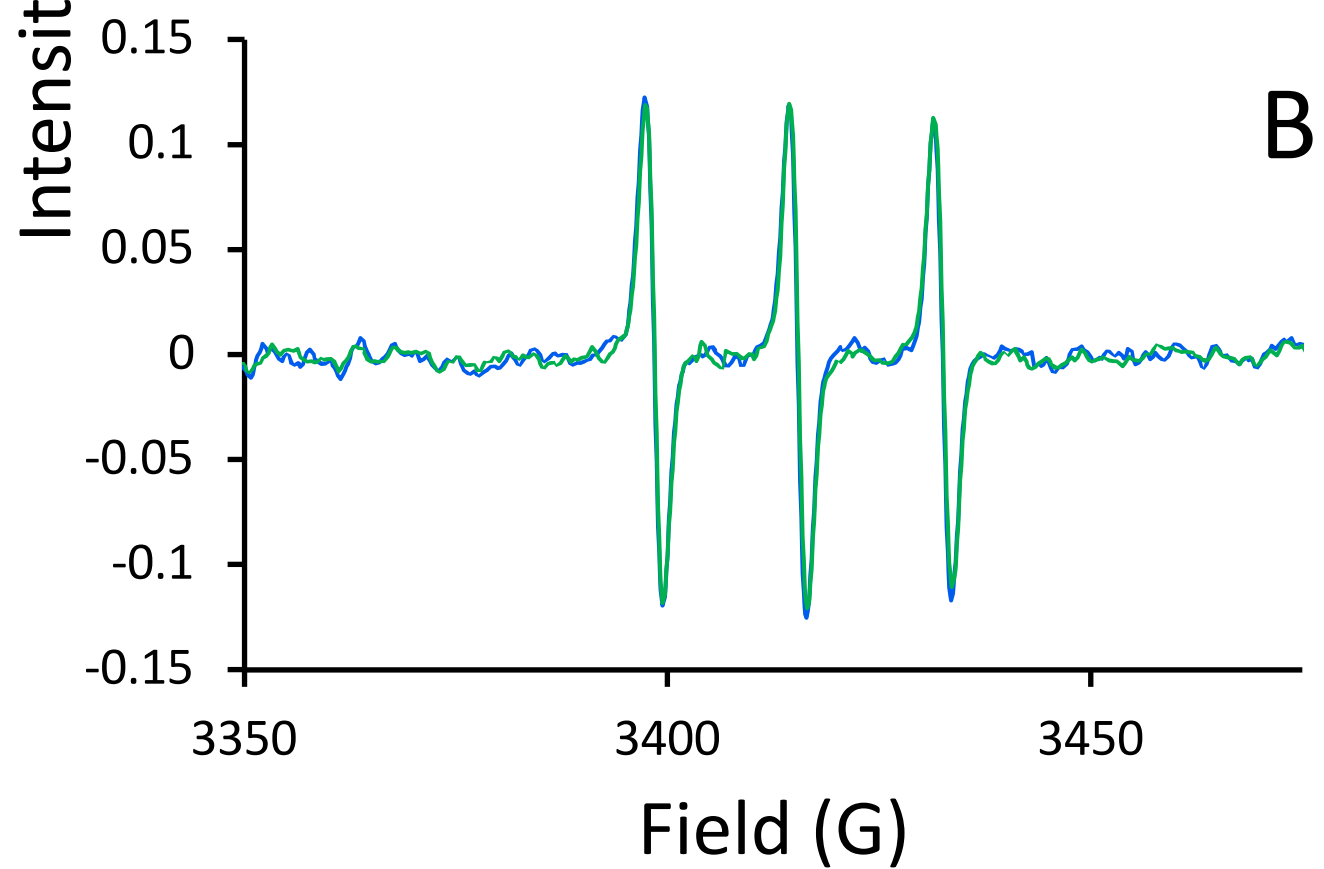

S1 Figure

Supplement: S1 Fig — The ESR spectra of A) TEMPOL alone (black trace), TEMPOL with SARS-CoV-2 Nsp9 + 2NSP90 (red trace), and B) TEMPOL with SARS-CoV-2 Nsp9 + 2NSP23 (green trace), TEMPOL with SARS-CoV-2 Nsp9 (blue trace) superimpose very well, confirming the invariance of the nitroxide dynamic regime in the presence of the proteins and hence the absence of specific tight interactions, consistently with the τc values reported in the main text that were calculated from signal spacings and amplitudes [18–20]. The trace overlay was split into two panels to avoid graphic crowding. The concentration of any species was always 10 μM. (PDF) [file pone.0303839.s001.pdf]

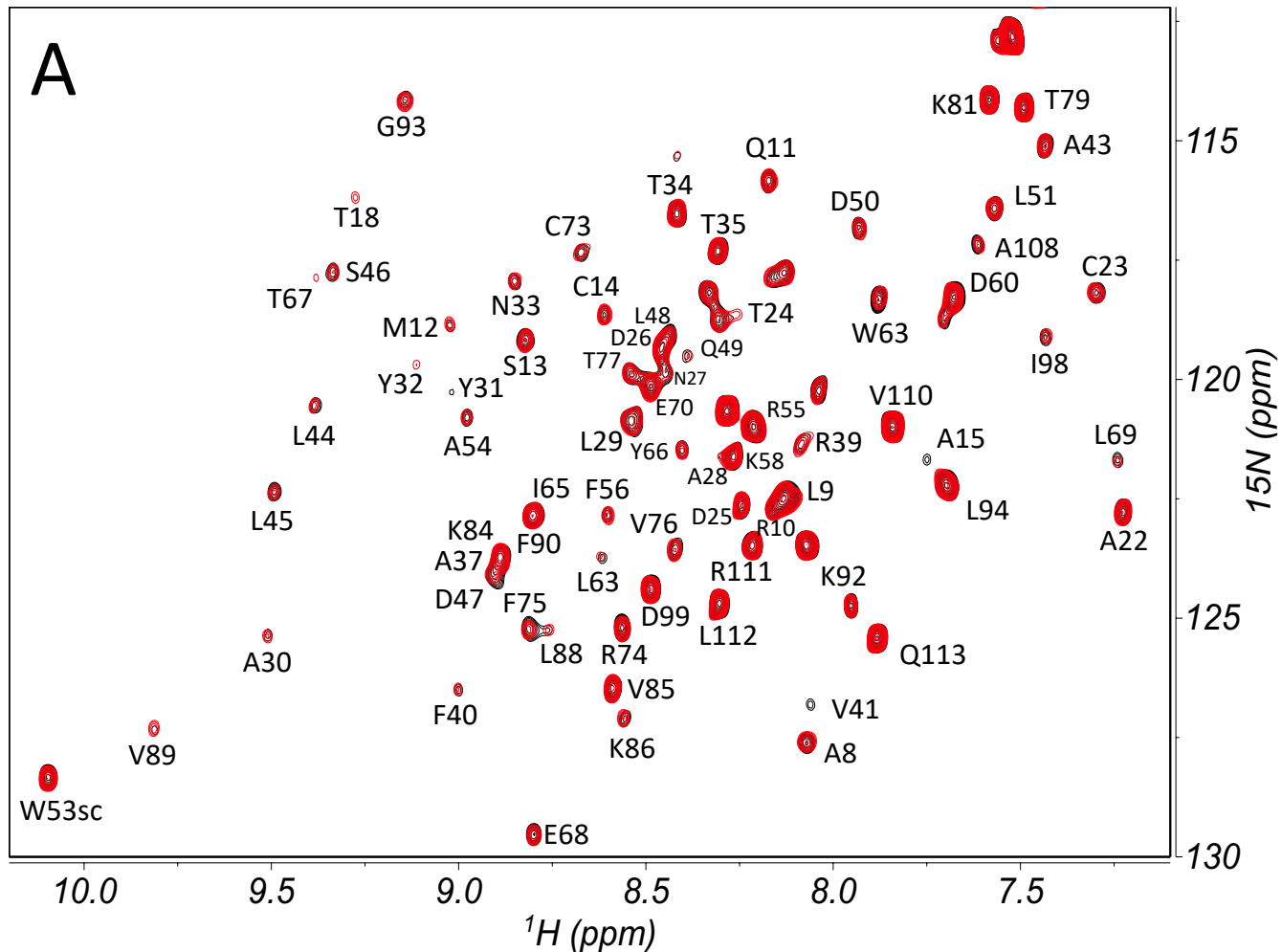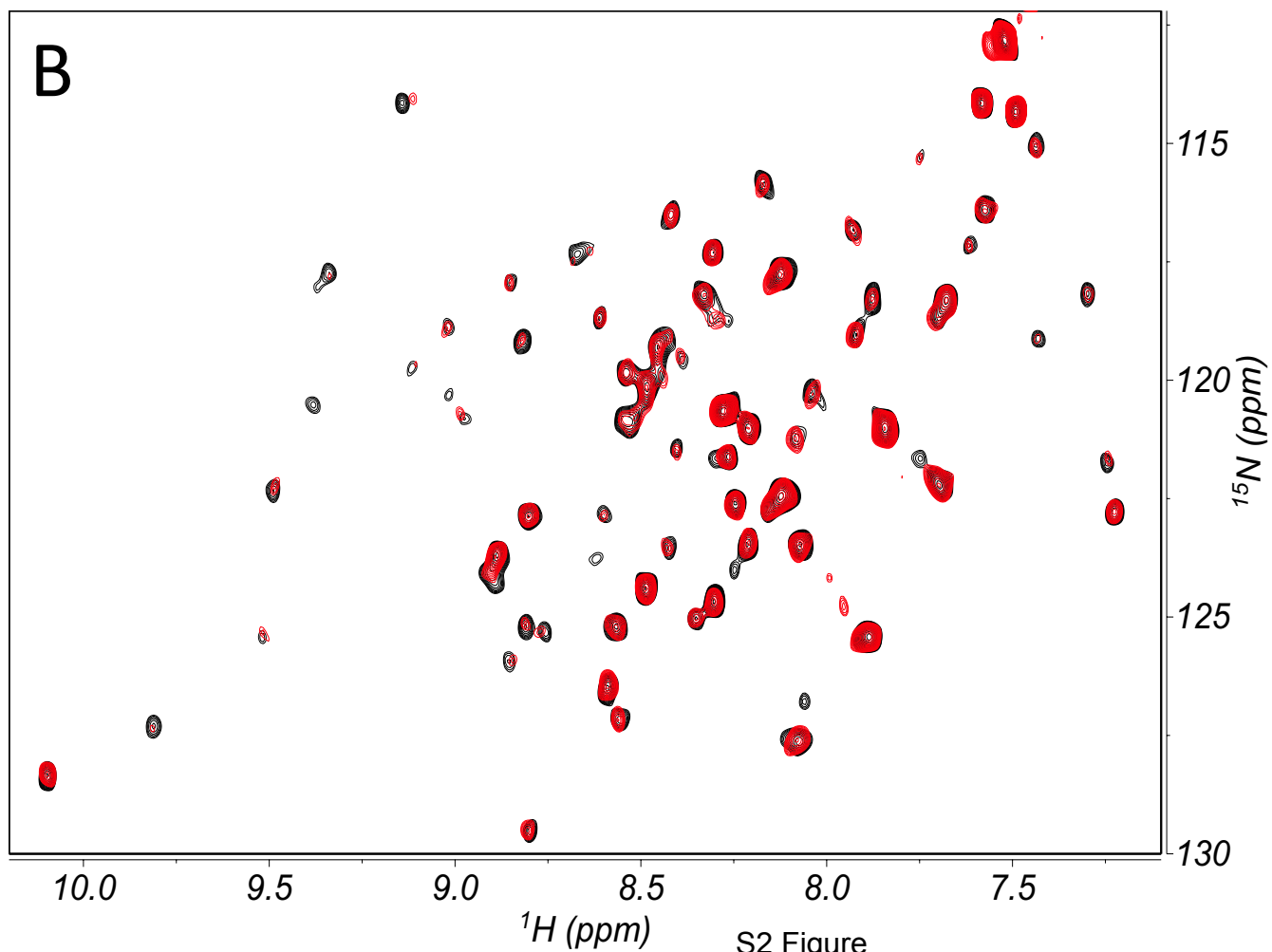

Supplement: S2 Fig — 15N−1H HSCQ spectra overlay of labeled SARS-CoV-2 Nsp9 without (A) and with (B) unlabeled 2NSP90, in the absence (black contours) and presence (red contours) of TEMPOL paramagnetic perturbation. The assignments of the backbone NHs (and W53 side chain) [26] are also reported. Data collection was carried out at 298 K on 18 μM Nsp9 alone or with 5.6 μM 2NSP90, before and after addition of 1 mM TEMPOL, with a relaxation delay of 0.3 s, according to the PENELOP protocol for off-equilibrium acquisitions [18]. The contour plot pairs with and without TEMPOL are always drawn at the same vertical scale, whereas a two-fold scale increment is applied to panel B data (with 2NSP90) compared to panel A data (without 2NSP90). (PDF) [file pone.0303839.s002.pdf]

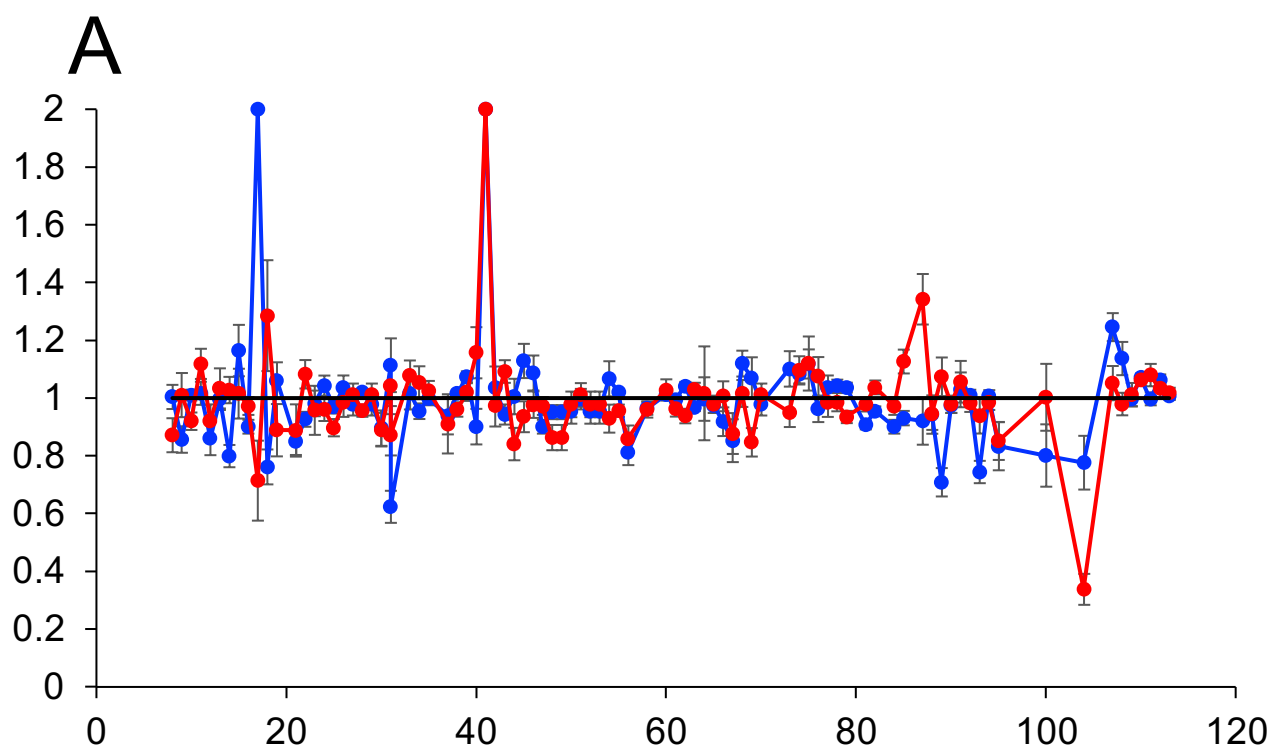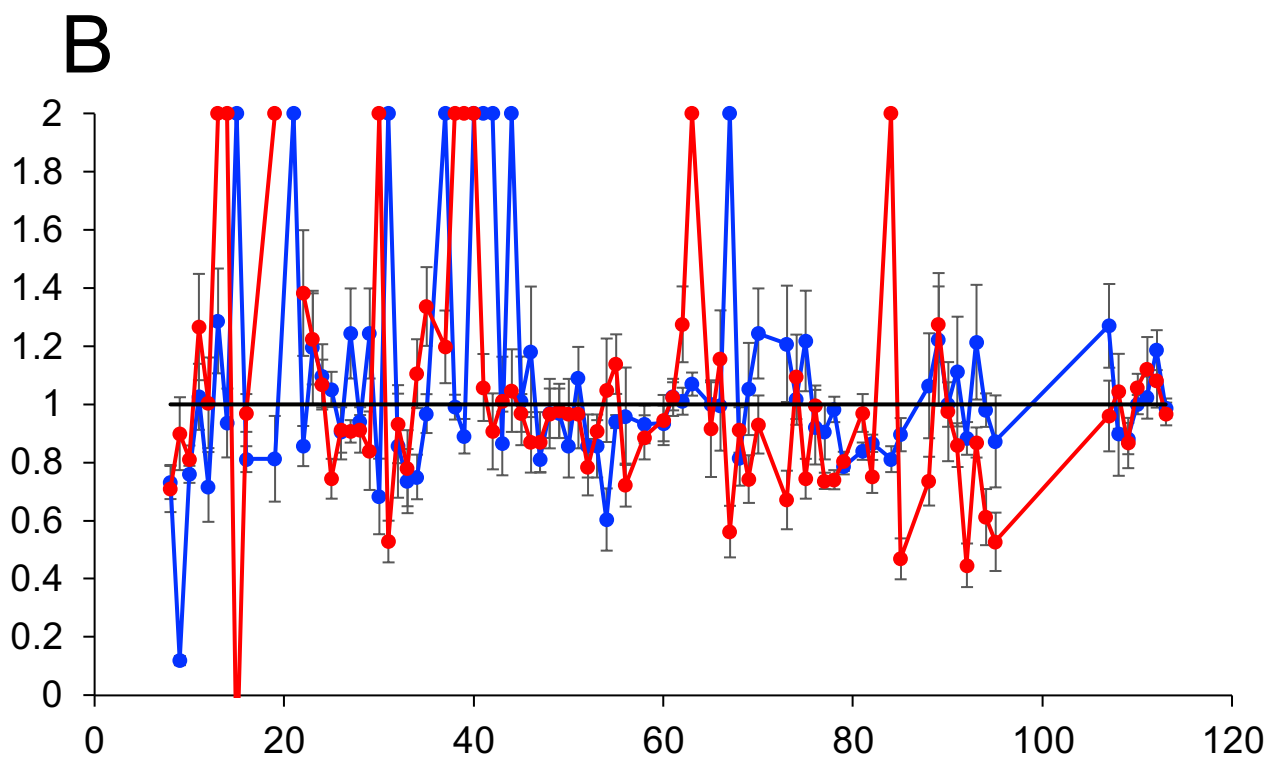

S3 Figure

Supplement: S3 Fig — Overlay plot of the AN values obtained from 1H-15N HSQC spectra of 18 μM SARS-CoV-2 Nsp9 in the absence (A) and presence (B) of 5.6 μM 2NSP90 as determined by 1.0 mM Tempol at 298 K with a relaxation delay of 0.3 s (blue, nonequilibrium condition) and 3 s (red, equilibrium condition). The data for segments and residues 1–7, 20, 36, 59, 87, 96–106, and the relative abscissa points are not reported because of the absence of the corresponding signals from the spectra. The locations of prolines (devoid of NH) are also skipped on the abscissa axis. (PDF) [file pone.0303839.s003.pdf]

S4 Figure

A

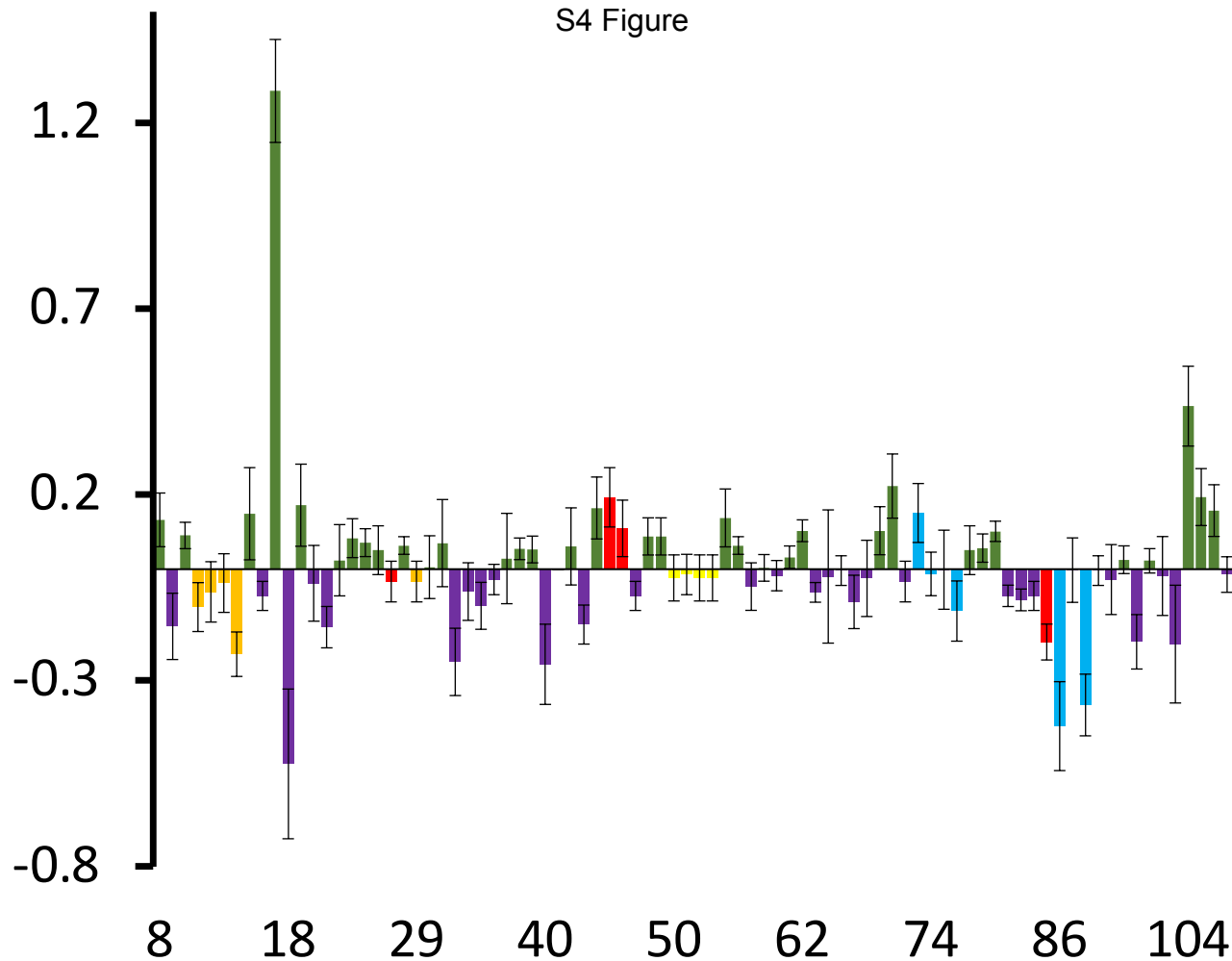

B

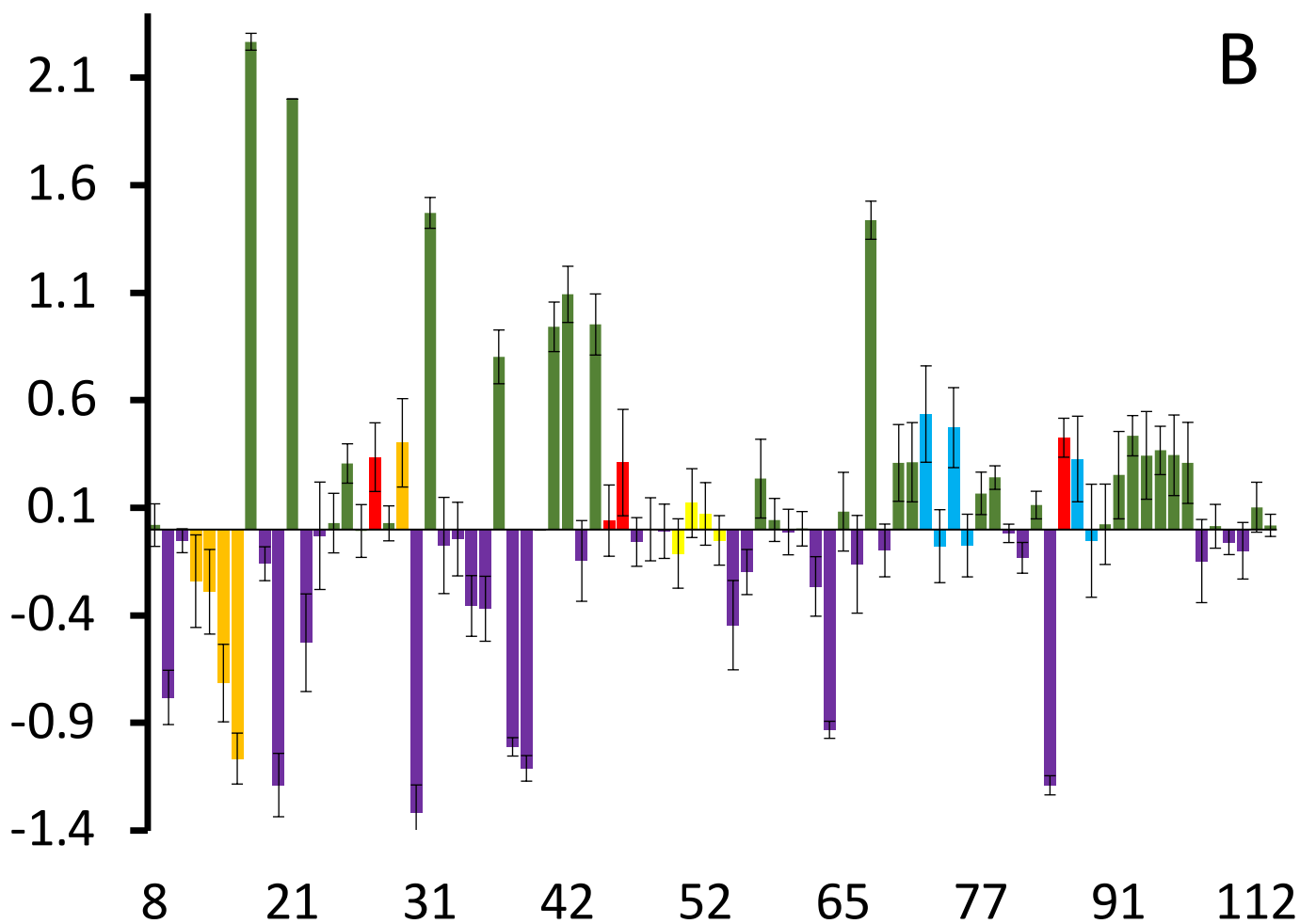

Supplement: S4 Fig — Bar plot of {AN[off] − AN[eq]} differences highlighting the locations of the Type I pattern (green bars) and Type II pattern (purple bars) for 18 μM Nsp9 alone (A) and in the presence of 5.6 μM 2NSP90 (B). Same plot as Fig 3 of main text without expansion truncation. Refer to Fig 3 caption for color code and other information. (PDF) [file pone.0303839.s004.pdf]

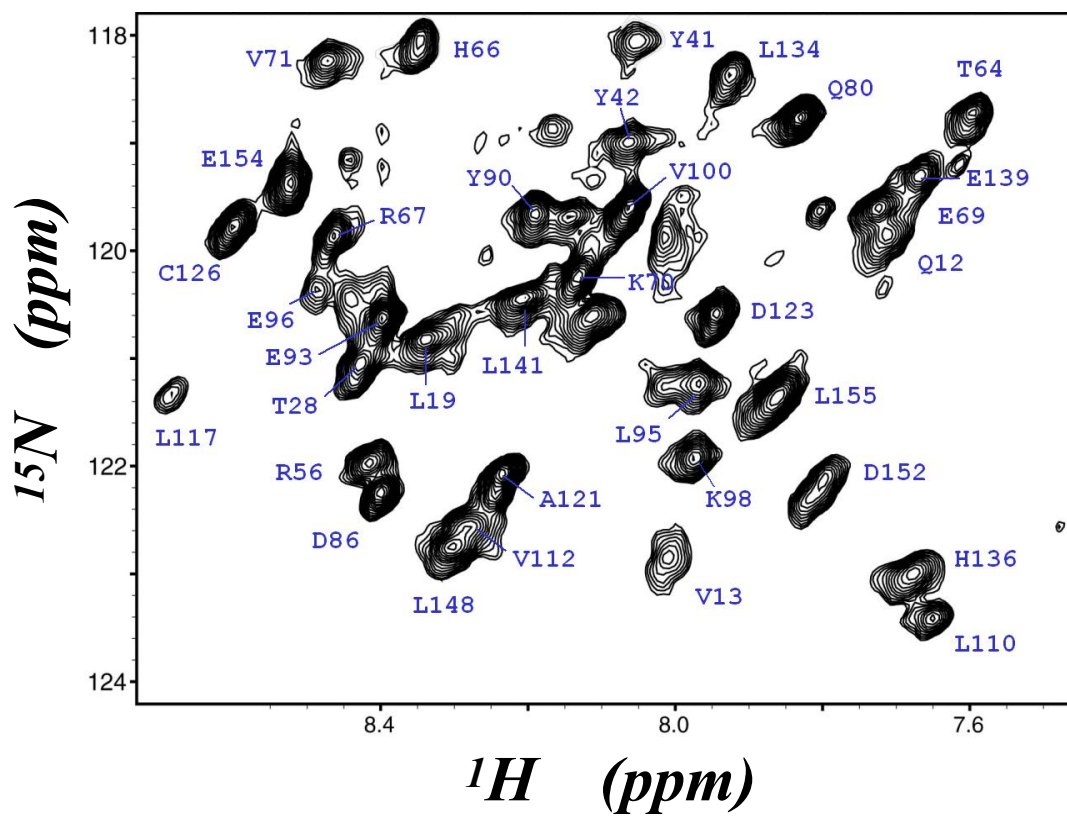

S5 Figure

Supplement: S5 Fig — 15N-1H HSQC spectrum U (15N, 13C) 70% 2H EMILIN1 C1q domain, a 52 kDa homotrimer. The spectrum was obtained at 17.6 T (750 MHz 1H frequency) and 310 K. The most crowded spectral region shown in the map benefits from 70% 2H labeling, but a lower resolution spectrum is also observed with the fully protonated sample. (PDF) [file pone.0303839.s005.pdf]
