# Supplementary material for: Assessing nanobody interaction with SARS-CoV-2 Nsp9
Source: PLoS One. 2024 May 17;19(5):e0303839. doi: 10.1371/journal.pone.0303839 (PMC11101046; doi:10.1371/journal.pone.0303839)
Supplement: S1 Table — (PDF) [file pone.0303839.s006.pdf]

**S1 Table. Peak loss and attenuation in $^{15}N$-$^{1}H$ HSQC spectra of SARS CoV-2 Nsp9 titrations with nanobodies.**

| | Peak loss | |
|---|---|---|
| **Nanobody : Nsp9 ratio** | **2NSP23[(a)]** | **2NSP90[(a)]** |
| 0.17:1 | - | - |
| 0.32:1 | - | *β4 (W53) l3-4 (50)* |
| 0.43:1 | *β1 (17) β3 (40, 46) β4 (52) β5 (67) β7 (89)* **l***5-6 (69)* | *β2 (28) l5-6 (69)* |
| 0.54:1 | *β1 (12, 14) β2 (30, 31, 32, 33, 35) β4 (53, 54, 56) β5 (64, 66) β7 (87, 88) α1 (108) l1-2 (18, 19, 23) l3-4 (50) l7-α1 (91, 93)* | *β1 (12,17) β2 (30, 31, 32, 35) β3 (40, 41, 44, 45) β4 (52, 54, 56) β5 (67, 68) β7 (88, 89) α1 (108) l1-2 (18, 23) l7-α1 (93)* |
| 0.63:1 | *β1 (11, 13, 15) β2 (27, 29, 34) β3 ( 44, 45) β5 (65, 68) β6 (73) β7 (86, 90), α1 (95) l1-2 (22), l2-3 (37) l4-5 (58, 60, 62), l5-6 (70)* | *β1 (11, 13, 15) β2 (27, 29, 33, 33sc 34) β3 ( 38, 46) β4 (53sc) β5 (64, 66) β6 (74) α1 (95) l1-2 (19, 21, 22, 24) l2-3 (37) l4-5 (62, 63) l5-6 (70) l7-α1 (91)* |
| TOTAL | *β1 (***6/8***), β2 (***8/10***), β3 (***4/9***), β4 (***4/6***), β5 (***5/5***), β6 (***1/7***), β7 (***5/9***), α1 (***2/15***) l1-2 (***4/8***), l2-3 (***1/2***), l3-4 (***1/5***), l4-5 (***3/6***), l5-6 (***2/4***), l7-α1 (***2/4***)* | *β1 (***5/8***), β2 (***9/10***), β3 (***6/9***) β4 (***4/6***), β5 (***4/5***), β6 (***1/7***), β7 (***2/9***), α1 (***2/15***) l1-2 (***6/8***), l2-3 (***1/2***), l3-4 (***1/5***), l4-5 (***2/6***), l5-6 (***2/4***), l7-α1 (***2/4***)* |

| Fastest peak attenuation [(b)] | |
|---|---|
| **2NSP23** | **2NSP90** |
| *14, 17, 18, 19, 30, 32, 40, 46, 52, 54, 56, 67, 69, 89, 93, 108* | *13, 30, 31, 33, 41, 43, 51, 53, 54, 56, 67, 69, 108, 111* |

(a) The progressive loss of Nsp9 backbone or side-chain NH signals upon nanobody titration is reported with the indication, in parenthesis, of the residue number (italics) and the secondary structure location (β, α and *l* are strand, helix and intervening loop). The TOTAL rows report the counts of residue signals that are lost out of total number of residues in each secondary structure elements (numbers are in bold to avoid confusion with residue sequence numbers). The relative titrations were carried out at 278 K (2NSP23) and 276 K (2NSP90) to slow down the peak losses that were much more massive and fast at ambient temperature.

Although quite similar, the relative interactions of the two nanobodies exhibit slight differences for the involved epitopes, with 2NSP23 addressing a more extended surface on β7 strand, balanced in 2NSP90 interaction by small extensions of 1-2 loop and β3 strand. These differences map to epitopes e2 and e4 that are adjacent on each Nsp9 monomer contacted by 2NSP23 or 2NSP90 (see Fig. 3 of main text). Nsp9 sequence is reported below with the location of the secondary structure elements along the sequence highlighted in turquoise (β-strand) and yellow (α-helix). In addition, the nanobody sequences are also reported with the indication of the complementarity determining regions, CDR1 (blue), CDR2 (green) and CDR3 (red).

(b) The residue numbers (italics) with fastest attenuation upon nanobody titration are listed. The peaks with the steepest decrease in intensity were identified when the slope of their relative intensity attenuations was larger than the average attenuation slope increased by one standard deviation.

```
         10         20         30         40         50         60
NNELSPVALR QMSCAAGTTQ TACTDDNALA YYNTTKGGRF VLALLSDLQD LKWARFPKSD
             β1                   β2         β3                β4
         70         80         90        100        110
GTGTIYTELE PPCRFVTDTP KGPKVKYLYF IKGLNNLNRG MVLGSLAATV RLQ
    β5          β6          β7               α1
```

2NSP23:

```
         10         20         30         40         50         60
QVQLQESGGG LVQPGGSLRL SCAASGLAFS MYTMGWFRQA PGKEREFVAM IISSGDSTDY
         70         80         90        100        110        120
ADSVKGRFTI SRDNGKNTVY LQMDSLKPED TAVYYCAAPK FRYYFSTSPG DFDSWGQGTQ
        130        140
VTVSSAAAYP YDVPDYGSHH HHHH
```

2NSP90:

```
         10         20         30         40         50         60
QVQLQESGGG LVQTGDSLRL SCAVSGRTFS TYSVGWFRQA PGKEREFVAL RWSGGTTYYA
         70         80         90        100        110        120
DSVVGRFTVS RDNAKNTVYL EMNSLKPEDT AVYYCAADRG SGSYSPTYRW DYWGQGTQVT
        130        140
VSSAAAYPYD VPDYGSHHHH HH
```
