# Supplementary material for: Assessing nanobody interaction with SARS-CoV-2 Nsp9
Source: PLoS One. 2024 May 17;19(5):e0303839. doi: 10.1371/journal.pone.0303839 (PMC11101046; doi:10.1371/journal.pone.0303839)
Supplement: S1 File — (PDF) [file pone.0303839.s007.pdf]

S1 File. Normalized attenuation (A_N) and relative error definitions.

A_N values were calculated according to:

$$A_N^k = \left(2 - \frac{\iota_p^k}{\iota_d^k}\right)$$

where the running index *k* refers to the *k*th residue amide signal and the $\iota_{p,d}^k$ values are the corresponding auto-scaled intensities of each signal in the presence (subscript *p* for paramagnetic) and absence (subscript *d* for diamagnetic) of paramagnetic probe, defined as:

$$\iota_{p,d}^k = \frac{I_{p,d}^k}{\frac{1}{n}\sum_{k=1}^{n} I_{p,d}^k}$$

with *n* being the total number of measured signals and *I* denoting the signal intensities. From the latter equation, it is seen that the scaling factor is simply the mean value over the *n* residues whose signal intensity can be estimated. The mean value of the individual auto-scaled intensities ($\iota_{p,d}^k$) is thus unitary, by definition [18-20]. Therefore, values of A_N above or below unity identify larger or smaller attenuations, respectively, with respect to the normalized average attenuation.

From the previous definitions, the error on the individual A_N values can be calculated as:

$$\Delta A_N^k = A_N^k \times \sqrt{\left[\frac{\Delta I_p^k}{I_p^k}\right]^2 + \left[\frac{\Delta I_d^k}{I_d^k}\right]^2 + \left[\frac{\frac{1}{n^2}\Sigma\left(\Delta I_p^k\right)^2}{\left(I_p^{av}\right)^2}\right] + \left[\frac{\frac{1}{n^2}\Sigma\left(\Delta I_d^k\right)^2}{(I_d^{av})^2}\right]}$$

where the first two terms under the square root represent the error on the relative intensity value of the *k*th residue signal, that is, the signal intensity ratio in the presence and absence of nitroxide, the *ΔI* are the experimental intensity uncertainties obtained from the individual peak signal-to-noise value, and the superscript *av* stands for average.
