# Supplementary material for: Assessing nanobody interaction with SARS-CoV-2 Nsp9
Source: PLoS One. 2024 May 17;19(5):e0303839. doi: 10.1371/journal.pone.0303839 (PMC11101046; doi:10.1371/journal.pone.0303839)
Supplement: Attachment — Submitted filename: Response_to_Reviewers.pdf [file pone.0303839.s008.pdf]

Reviewer 1

1.Introduction should contain about expirement question without writting about result and conclusion

All references to results and conclusions were eliminated from Introduction.

2.References format should be maintained as per journal in materials and methods

The references reported twice in the text with proper and erroneous formats were corrected.

3.Justify your results listed in methodology .

There is no methodology section listing our results in the main text, nor in Supplementary Information. We do not understand what the reviewer means.

4.Discussion should be comparative with the obtained results and avaiable data about the study

The Discussion section the reviewer read in the first version of the manuscript was divided into two parts addressing the Nsp9 complex stoichiometry and the alternative structure recently reported by Pan and colleagues (PLoS ONE 2023;18(4): e0283194). Both subsections already consisted of comparisons of different stoichiometries and conformational options with the proposed composition and structural arrangement. These characteristics have been retained in the current version of the manuscript. Therefore the reviewer requests are implemented.

5.Conclusion must include furthur research and progression that can be done with the above research along with their applications.

We appreciate the suggestion of the reviewer. We added to the conclusions of the revised manuscript additional statements concerning the development of the research for possible applications.

Reviewer 2

The PENELOP technique itself seems sound (has been published by the same group - no citations so far though - in well known and reputable journals (Analytical Chemistry ref 18 and PCCP ref 19) and the analysis of the technique in ref 19 in particular is good. The conclusions they draw from the technique wrt exposed surfaces and buried surfaces of the protein in the presence of the nanobody are compelling. Conclusions related to exchange dynamics are a bit less so - ref 19 says this is a reasonable explanation but stops short of saying this is the only explanation - but it seems to only be a passing mention in the paper. Needs to be validated

We thank the reviewer for appreciating our work on paramagnetic perturbation of protein spectra. Based on the reviewer comment, that appreciation is only partial, however. The reviewer in fact seems to cast doubts on the interpretation of Type II pattern of PENELOP method when remarking the absence, in our previous paper, of an explanation other than slow-to-intermediate exchange dynamics. This statement is not accompanied by any of the possible alternative explanations the reviewer implicitly hints at and/or seems to glimpse, but a lapidary “Needs to be validated”.
The works dealing with the subject (listed as refs. 18, 19, 20) were published in reputable journals after passing regular peer review processes and, within the context of the discussed applications, no objection concerning possible alternative explanations was ever raised.
Reference 19, the one specifically cited by the reviewer, describes at length the basis of our interpretation providing both an independent experimental reproduction of the exchange dynamics mechanism (see Fig. S6 in Supplementary Information and page 6252 of reference 19), and an extensive validation of the results obtained from the proposed interpretation of equilibrium and off-equilibrium paramagnetic attenuation. The validation consists of independent and systematic measurements of relaxation dispersion and $T_{1\rho}$ at different spin-lock field strengths of the same molecular group signals that were considered for equilibrium and off-equilibrium paramagnetic perturbation. Therefore our conclusions related to exchange dynamics have already been thoroughly validated.

The Figure 1 presents off-equilibrium HSQC spectra alone. Having the equilibrium HSQC spectra in addition to (or instead of) off-equilibrium spectra would be more useful to emphasise the conclusions they draw in that section (they say that they present raw A_N values in figure S2 of the supplementary information but I can't see this figure)

Figure 1 (renumbered Figure 2 in the revised version to comply with the requests of another reviewer) has been modified according to the reviewer suggestion and the comparison of the equilibrium HSQC maps in the absence ad presence of 2NSP90 is now reported. The corresponding comparison under non-equilibrium conditions is shown in Supplementary Information (Figure S2) to avoid extending further the manuscript (again to comply with the request from another reviewer). Upon resetting the HSQC overlays to prepare the new figure, we realized that the previously reported superpositions had been erroneously selected by inadvertently swapping the correct spectral maps with unrelated ones occurring in the same experiment directory. The mistake has been corrected in Figure 1 (and Figure S2), along with the relative main

text comment to the same figure. The revision also enabled correcting erroneously reported information on residues 86 and 87. In addition, the reviewer mentions a missing Supplementary Information figure, namely Figure S2 (renumbered as Figure S3 in the revised version and reported below with the corresponding caption). We apologize for the inconvenience. We do not understand why that figure was missing. In the manuscript downloaded from the journal site, the final link to the Supplementary Information correctly retrieved the file and the figures therein. We hope the revised manuscript version will not present the problem highlighted by the reviewer.

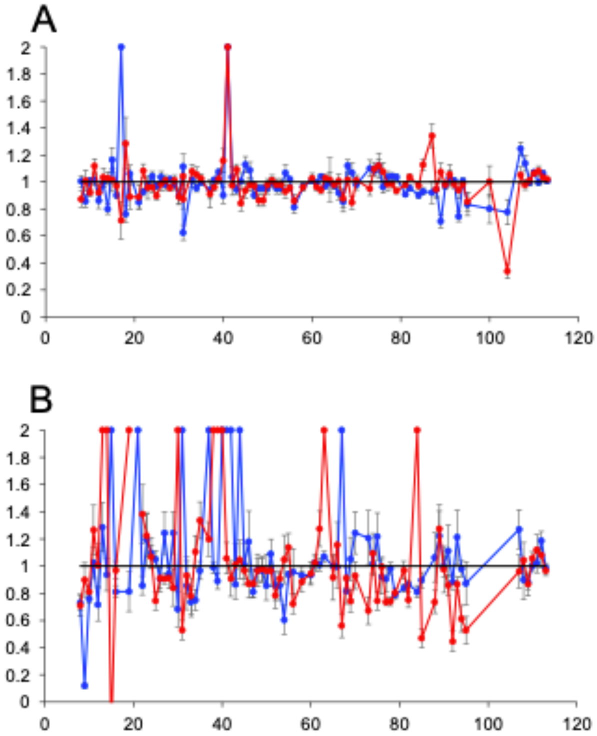

S3 Figure. Normalized attenuation (A_N) pattern of Nsp9 – 2NSP90 system. Overlay plot of the A_N values obtained from ^1H-^{15}N HSQC spectra of 18 μM SARS-CoV-2 Nsp9 in the absence (A) and presence (B) of 5.6 μM 2NSP90 as determined by 1.0 mM Tempol at 298 K with a relaxation delay of 0.3 s (blue, nonequilibrium condition) and 3 s (red, equilibrium condition). The data for segments and residues 1–7, 96–106, 20, 36, 59 and 87, and the relative abscissa points are not reported because of the absence of the corresponding signals from the spectra. The locations of prolines (devoid of NH) are also skipped on the abscissa axis.

While the methodology is nifty, the conclusions can further validated . In both the papers, the antiviral activity has not been demonstrated. If the nanobodies work across strains again, the potency not demonstrated.

The reviewer furtherly mentions validation, this time concerning the antiviral activity of the nanobodies. The current manuscript only deals with the *in-vitro* study of the interaction between Nsp9 and one of the previously reported nanobodies (2NSP90), much like the previously published works on the subject, i.e. Esposito et al., Adv. Biol. 2021; 5 (12):2101113; and Pan et al., PLoS ONE 2023;18(4): e0283194, numbered 12 and 21 in our reference list. The conclusions of all the previous *in-vitro* characterizations suggested a possible application of the tested nanobodies for antiviral therapy. The manuscript under consideration reaches the same conclusions using additional, different evidence. However, as reported in the conclusion section of the revised version, a successful *in-vivo* test of the antiviral properties of the addressed nanobodies was recently obtained. Therefore the antiviral activity of the nanobodies has indeed been demonstrated.

Reviewer 3

This study is basically an extension of a previous study by the same authors (Esposito et al, Adv Biol 5(12):2101113; 2021) where the nanobody was isolated and characterised by 2D NMR spectroscopy and chemical shift analysis.

Results of this work includes a very limited amount of new experimental data (PENELOP protocol, previously established, based on NMR paramagnetic perturbation) in addition to new molecular dynamics simulations. The authors confirmed their previously published NMR data in terms of stoichiometry and epitope mapping.

The very comprehensive discussion section contains a comparison to a recently published structure by X-Ray crystallography (Pan et al, PLoS One 18(4): e0283194; 2023) revealing significant differences, in particular no significant structural rearrangement is observed in the present study compared to the crystal structure.

Overall, the experimental execution is solid, and the conclusions supported by the data, however I have significant reservations about the novelty of this manuscript. A very limited amount of new data is presented, and the data basically just confirm a model that is already published in the author's earlier work.

We thank the reviewer for appreciating the solidity of our work. We do not agree with the reviewer's concern about the limited novelty of our findings. It is in fact necessary to take into account the circumstances that prompted us to carry out the measurements that are presented in the manuscript. In march 2022 we were contacted by Dr. Littler, the corresponding author of the paper by Pan *et al.* (PLoS ONE 2023;18(4): e0283194), as mentioned in the former cover letter of our submission. He proposed a collaboration based on their crystallographic results. We replied we could accept the collaboration if a section comparing the different results could be included in the manuscript. We also sent a draft for that section but no longer heard from Dr. Littler until his paper came out. As it can be easily verified, the work by Littler's group barely mentions our previous conclusions. The same work also reports an NMR titration for which a coarsely wrong interpretation is proposed that obscures the already published and discussed significance of our (and their) titration, thereby ruling out any discussion of the experimental observations in solution. Therefore, the manuscript which is now being considered is the product of further efforts of our group to provide alternative and additional evidence challenging our previous results in comparison with those deriving from an authoritative technique such as X-rays. As a matter of fact, the new evidence support our former interpretation.
As well known, in the presence of a dispute, PLoS ONE policy entails submitting the disputing manuscript to the author of the disputed paper. This procedure has been accepted and applied for our manuscript too. It is worth to point out that the irreprensible policy PLoS ONE adopted with our manuscript was not applied for the PLoS ONE manuscript by Dr. Littler and colleagues who expressed and studied the complex of Nsp9 with a nanobody that was first published by us reaching different conclusions. We do not see any fault of the journal nor the reviewers because the difference with respect to the previous results reads softened or misinterpreted in the paper by Dr.

Littler and colleagues. Indeed, one cannot expect the Editor or the reviewers to examine in detail all the quoted literature.

At the very least, a detailed comparison between the model presented and the published crystal structure is required and a set of Nsp9 mutants should be designed to either confirm or exclude the model presented in this study.

Also, have effort being made to crystallise the presented Nsp9-nanobody complex? This is not mentioned in the manuscript.

Our manuscript reports the description of the differences between the model we propose and the crystal structure recently published by Pan and coworkers (PLoS ONE 2023;18(4): e0283194). In their paper those authors show a quite detailed comparison between their peculiar monomer and dimer structures and the typical Nsp9 monomer and dimer arrangements found in any previous crystal structure of the protein. The tetrameric assembly that we propose to be targeted by 2NSP90 and 2NSP23 corresponds to the crystallographic structure published by Zhang and colleagues (Mol. Biomed. 2020; 1:5, reference 39 in our manuscript) which exhibits both inter-helix and inter-β-sheet interfaces. The details of that assembly are found in the original work by Zhang *et al.* (ref. 39) and in references 40-45 of our list. In our manuscript the most salient features are however comparatively highlighted. To limit the length of the work, as requested by other reviewers, we omit additional some details that can be recovered from the quoted literature.
Except for the mutant with Cys14, Cys23 and Cys73 replaced by serines, we did not test other Nsp9 variants. A systematic investigation involving Nsp9 mutants could provide evidence on the mapped epitope composition and uniqueness, although the repertoire of the collected nanobodies is quite large. The 136 earlier-reported nanobody sequences that were selected by ELISA test (see our reference 12) bear 40 diverse CDR3 which may well extend the range of recognized motifs. On the other hand, using a different methodology to verify our former conclusions appeared a preferable option as it could allow a more focused test and hence a more direct reply to the result published by Pan and colleagues.

We did no attempt to crystallize the Nsp9-2NSP23 or Nsp9-2NSP90 complexes. Pan and colleagues report quite harsh crystallization conditions that were defined "non-physiological" and might have determined the profound structural change undergone by Nsp9.

Reviewer 4

In the work titled “Assessing nanobody interaction with SARS-CoV-2 Nsp9”, Esposito et al combined PENELOP, a paramagnetic perturbation methodology, and molecular dynamics simulations to further dissect the interaction between nsp9 and nanobody 2NSP90. NMPylation/RNAylation of Nsp9 and their roles in SARS-CoV-2 capping, which is being studied extensively. Besides the medical usage, nanobody can also be an extra tool for nsp9 studies. Mapping the binding sites and characterizing the binding features of nsp9 – nanobody is essential. Some concerns need to be addressed before being considered for acceptance.

We thank very much the reviewer for the meticulous reading of our manuscript and the precious suggestions. We are also pleased the reviewer shares our view on the relevance of Nsp9 epitope mapping.

Major:
1. The Discussion and Conclusion sections are too long, which makes it hard to catch the main point. Importantly, it’s difficult to see the new findings from this study.
2. Page 11 lines 14 – 20: please provide a schematic to illustrate the Type I and Type II patterns for a broader reader; Figure 3: Please provide a rotated structure view to show the analogous epitope ensemble; S1 figure doesn’t match the results on page 11 line 22. Only TEMPOL alone and TEMPOL + nsp9 + 2NSP90 are shown.
3. Please make the NMR data publicly available.
4. For discussing the increased Type II pattern of e1, the authors used “translating into an additional interaction exchange” on page 15 – line 5. But what does it mean? Even as stated on page 19 line 19 “loose enough to preserve the Nsp9 surface accessibility for the paramagnetic probe”, it still doesn’t explain the increased Type-II pattern. Is it because the nanobody disrupts the interdimer domain?

1.The Conclusions and the Discussion sections were partially shortened. In fact other reviewers asked to discuss in detail the comparison between our results and the literature evidence which we did essentially in the Discussion section. As for the novelty of the findings, it is necessary to take into account the circumstances that prompted us to publish the results that are presented in the manuscript. In march 2022 we were contacted by Dr. Littler, the corresponding author of the paper by Pan *et al.* (PLoS ONE 2023;18(4): e0283194), as mentioned in the former cover letter of our submission. He proposed a collaboration based on their crystallographic results. We replied we could accept the collaboration if a section comparing the different results could be included in the manuscript. We also sent a draft for that section but no longer heard from Dr. Littler until his paper came out. As it can be easily verified, the work by Littler’s group barely mentions our previous conclusions. The same work also reports an NMR titration for which a coarsely wrong interpretation is proposed that obscures the already published and discussed significance of our (and their) titration, thereby ruling out any discussion of the experimental observations in solution. Therefore, the manuscript which is now being considered is the product of further efforts of our group to provide alternative and additional evidence challenging our previous results in comparison with those deriving from an authoritative technique such as X-rays. As a matter of fact, the new evidence support our former interpretation.

2.Schematics are not allowed by PLoS ONE style. Therefore a new figure (Figure 1) has been introduced to illustrate Type I and Type II patterns. All the other figures have been renumbered accordingly.
In the revised version, Figure 3 (now Figure 4) also includes the rotated structure that shows the epitope distribution on the opposite side of the tetramer.
To avoid graphic crowding, Figure S1 did not report all the mentioned ESR traces but only the most relevant ones, namely those of isolated Tempol and in presence of Nsp9 +2NSP90. An additional panel was added in Figure S1 showing the overlay of Tempol + Nsp9 (blue trace) and Tempol +Nsp9+2NSP23 (green trace).

3.The NMR data are available according to the Editor policy. The data were deposited at Dryad repository and are accessible at URL: https://doi.org/10.5061/dryad.905qfttsj . Prior to publication private access is possible at URL:
https://datadryad.org/stash/share/B87t_0iww9GjEnh6Ntq_BL_uZCxCdW-4U2uCc_Vr6x0.

4.The Nsp9 paramagnetic attenuation that is observed in Figure 3A (formerly Figure 2A) shows a consistent clear Type II pattern for the epitope region labeled e1 (Q11, M12, S13, C14, L29). Type II pattern identifies the occurrence of local exchange processes on the μs-ms time scale which entail relaxation contributions and are highlighted by a paramagnetic probe. Those relaxation contributions are responsible for an accelerated signal recovery leading to a decreased normalized attenuation under non-equilibrium conditions with respect to the corresponding equilibrium attenuation (the attenuations are always normalized with respect to the average attenuation). The local exchange processes can be structural fluctuations or intra/intermolecular exchange interactions taking place over slow-to-intermediate time scales. In the presence of 2NSP90 (Fig. 3B), the Nsp9 paramagnetic attenuation in the e1 region of the N-terminal segment shows a largely increased extent of Type II pattern, whereas residue L29 exhibits a clear Type I pattern. This Type I pattern indicates a hampered accessibility, as expected for an epitope region screened by the paratope of the nanobody, whereas the increase of Type II pattern at the N-terminal segment of Nsp9 should be the consequence of an additional exchange due to the nanobody that contacts region e1 with a loose binding. The statement "translating into an additional interaction exchange" (now modified as "translating into an additional exchange") thus describes such a scenario.
Along the same line it is possible to understand the statement "loose enough to preserve the Nsp9 surface accessibility for the paramagnetic probe". As above mentioned, the decreased attenuation under non-equilibrium conditions is the effect of increased signal recovery if a paramagnetic agent can approach the location of the additional exchange processes. This is the case with the e1 epitope region of Nsp9 that retains the accessibility for the nitroxide because of a dynamic binding to 2NSP90.

Minor:
1. Page 3 line 14: please delete "the most interesting"; lines 16 – 20 can be rephrased into one shorter sentence.
2. Page 4 line 16: "contributing less than 1% of total mutations" might be because nsp7 – 10 are small.

3. Page 6 line 12: it's better to write "H2O/D2O 95/5 (vol/vol)"; Line 18: "Microliter aliquots" of what? Also, "1 mM nitroxide".
4. Page 7 line 12: delete "see". In general, please delete "see" from all the figure citations.
5. Page 9 line 5: what are the parameters? line 6: Which one has more numbers in the slightly different number scenario? Line 20: "k-th".
6. Page 10 line 5: "0.150 M" is used here, which is a different format from other places, such as page 9 – line 10. Please keep one format for it; Line 10: α-carbons (also page 16 – line 16); Line 11: "the dissociating subunits"; Line 13: delete "the simulation"; Line 15: what differences?
7. The citation format on page 9 – lines 15/18, and page 10 – lines 4/12, is not right, please correct them and check the whole manuscript.
8. Page 11 lines 1 – 5: please rephrase the sentences here to make it clear.
9. Page 12 lines 5 – 7: repeated words as in Figure 1 legend can be found in these sentences. Please rephrase to reduce redundancy.
10. Figure 2: please indicate Figure 2 is truncated at the beginning of the legend to avoid confusion; Please indicate V85 in the figure; And why do the authors use different x-axis scales?
11. Page 13 line 7: it's nicer to show the fragment/residues sequentially; line 20: missing a comma.
12. Page 14 line 2: "locations"; line 14: delete "see"; line 18: "histograms" is not the correct word here. One should use "bar graph".
13. Figure S3: in the legend, should it be "Same plot as Fig. 2 of main text"?
14. Page 15 line 3: "increasing"; line 4: which data support "stressed by the low nanobody/Nsp9 ratio"?
15. Page 18 lines 10 – 11: please rewrite "An intermediate exchange in fact broadens up to merging with the baseline the NMR signal linewidths of the exchanging species, thereby preventing detection." It's difficult to understand now.

1.The requested deletion on page 3, line 14, was done. The lines 16-20 of the same page were summarized as "Due to their small size, high solubility and folding stability, nanobodies are at the forefront of the evolving field of antibody therapeutics [7]."

2. Non-structural proteins 7, 8, 9, 10 account for 7.5% of the residues of SARS-CoV-2 polyprotein, but their mutations represent only 2.3% of the total SARS-CoV-2 mutations. For Nsp9, in particular, the reported mutations represent only 0.3% of the total, but the 113 residues of the protein account for 1.6% of the total polyprotein residues. The data come from reference 15 (reported at the end of line 16, page 4). Therefore, Nsp7-10 really exhibit a low mutation propensity compared to the other SARS-CoV-2 proteins.

3. The requested corrections were implemented. The microliter aliquots are referred to the nitroxide mother solution and the relative specification was added to the text.

4. "see" was deleted in all figure citations.

5. The parameters referred to on page 9, line 5, are the adjustable values for the various terms of the NAMD2 forcefield as well as for the simulation protocol. This is now explicitly indicated in

the text. The numbers of Na^+ and Cl^- were alternatively varied with an excess < 10% for any species. The correction at line 20 was added.

6. All the requested corrections were introduced. The difference (not differences) mentioned at line 15 is the difference between monomer-monomer and dimer-dimer association free energy. The text has been corrected accordingly.

7. The citation format imperfections were corrected.

8. The sentences on page 11, lines 1-5, were rephrased.

9. Redundancy was eliminated by rephrasing the text and Figure 2 (formerly 1) caption, according to the reviewer's suggestion.
It is necessary to add here that Figure 2 has been modified according to the request of another reviewer. Figure 2 now reports the comparison of the equilibrium HSQC maps in the absence ad presence of 2NSP90. The corresponding comparison under non-equilibrium conditions is shown in Supplementary Information (Figure S2) to avoid extending further the manuscript. Upon resetting the HSQC overlays to prepare the new figure, we realized that the previously reported superpositions had been erroneously selected by inadvertently swapping the correct spectral maps with unrelated ones occurring in the same experiment directory. The mistake has been corrected along with the relative main text comment. The revision also enabled correcting erroneously reported information on residue 86.

10. The suggestion of the reviewer has been added in the caption of the Figure (now 3 after renumbering). A red asterisk marks now the position of V85, as requested. Besides the residue signals that are missing both in presence and absence of 2NSP90 (listed in the caption), other three signals are not observed when the nanobody is present. This explains the different abscissa axes of panels A and B in Figure 3.

11. The residues/ fragments are now sequentially ordered in Figure 3 caption. The , missing comma on page 13, line 20, has been added.

12. "locations" and "bar graphs" replace now the previous text; "see" was deleted.

13. The caption of Figure S3 now reports "Same plot as Fig. 3 of main text" (because of renumbering Figure 2 became 3).

14. The suggested correction (increasing) has been introduced. As for the data that support the incidental statement "stressed by the low nanobody/Nsp9 ratio", there is the pattern reported in S1 Table that shows peak loss for the residues of e1 region at higher nanobody/Nsp9 ratio.

15. The sentence "An intermediate exchange in fact broadens up to merging with the baseline the NMR signal linewidths of the exchanging species, thereby preventing detection" has been changed into "An exchange in fact broadens the NMR signal linewidths of the exchanging species when its frequency matches the chemical shift differences of the involved species. The broadening leads to

flattening of the signals that merge with the baseline and become largely undetectable". We hope it will be easier to understand
