# Supplementary material for: Assessing nanobody interaction with SARS-CoV-2 Nsp9
Source: PLoS One. 2024 May 17;19(5):e0303839. doi: 10.1371/journal.pone.0303839 (PMC11101046; doi:10.1371/journal.pone.0303839)
Supplement: Attachment — Submitted filename: Response_to_Reviewers.pdf [file pone.0303839.s009.pdf]

Reviewer #5

We addressed all criticisms, requests and suggestions that were raised in the first round of the reviewers' scrutiny. This definitely helped to improve our manuscript, as acknowledged by all reviewers. We were a bit surprised on reading the further comments of Dr. Littler. The newly raised issues concern subjects that sometime can be included in the text as specific caveats to further improve it and adhere to the reviewer's requests. In the following, all new concerns are examined in detail.

My understanding is that there are several issues to consider. Coronaviral Nsp9 is predominantly a homodimer in solution, but its active form within the RTC is NiRAN-associated. However other cellular functions have been reported for Nsp9 which may utilise its homodimeric form. Secondly a free N-terminal amine on Asn-1 is now known to be an essential feature of this protein and is required for RNAylation activity. Many prior papers have recombinant "stubs" such as the authors current GAMG sequence, or my own GPG sequence for constructs used in Littler et al. 2020. This complicates the ability to draw physiologically relevant conclusions from stub-Nsp9 work.

The predominance of the Nsp9 homodimeric state in solution is clearly stressed in our manuscript, along with the monomeric state requirement for the formation of the viral replication and transcription complex (RTC). Following to the reviewer suggestion, the further information regarding the role of homodimers for other cellular functions was added in the Discussion (first subsection, pg. 18, line 10).
As for the role of the "stubs" we do not understand the point raised by the reviewer. Surely a free Asn1, i.e. without "stub", is necessary for the nucleotidylation of the N-terminal amine, a well-established event characterizing the role of Nsp9 in the RTC (Wang et al., Nucl. Ac. Res. 2021). However our work deals with the interaction of nanobodies with Nsp9. This interaction could be affected by the presence of polypeptide extentions preceding the N-terminal residue of Nsp9, because the local conformation might change thereby potentially biasing the interaction with the nanobody. There are, however, two arguments that Dr. Littler should consider. First, a few Nsp9 crystallographic structures have been obtained with constructs including "stubs" (e.g. 6w4b, 1uw7, 6wxd) that apparently did not introduce major conformational variations with respect to the sequence devoid of N-terminal extensions (e.g. 1qz8, 2J97, 3ee7, 7bwq, 2J98). Most meaningfully, all the X-ray structures exhibit a typically disordered N-terminal conformation involving the first 3-6 residues of the Nsp9 sequence, except some of those bearing a (declared) "stub" that apparently attenuates the N-terminal conformational dispersion. In solution, however, the presence of N-terminal elongations does not restrict the Nsp9 N-terminal mobility which was always observed to entail an intermediate conformational exchange with local loss of resonance detectability (Buchko et al., Biomol. NMR Assign. 2021;

Dudas et al., Biomol. NMR Assign. 2021; El-Kamand et al., Proteins 2022). We observed the same mobility and resonance loss pattern with either short (a single Met residue) and longer (GlyAlaMetGly) N-terminal extensions (Esposito et al., Adv. Biol. 2021). The second, much more stringent argument stems from the results we recently reported (Venit et al., BioRxiv 2023), as mentioned in ref. 52 of the manuscript under consideration. The anti-Nsp9 nanobody 2NSP23 inhibits viral replication by targeting Nsp9 in living cells. Hence, in spite of possible effects of the "stub" on the conformational mobility of the N-terminal region of the Nsp9 variants used to elicit the immune response or to study the selected nanobody interactions, the latter species targets successfully the natural Nsp9 sequence in SARS-CoV-2 infected cells. A statement concerning the possible bias introduced by the "stub" was anyway added at the end of the Conclusions section of the manuscript (pg. 25, lines 11-17).

In Esposito et al. 2021 Adv Biol the authors immunized llama with a triSer-Nsp9 mutant (C14S, C23S, C73S). I believe their own NMR suggests significant structural changes for this mutation as compared to wild type. Cysteines are unusual in that have both hydrophobic and polar characteristics. When they are buried within a hydrophobic pocket a serine mutant can be disruptive and structurally and in some instances an alanine mutation might work better. Indeed, most of the peaks lost correspond the changes seen in 8DQU. Any antibodies with this as immunogen could act to enforce whatever the triSer conformation is, which would be simple enough to crystalise as a control and check.

The statement of the reviewer about the "significant structural changes" of triSer-Nsp9 based on our NMR evidence has no foundation but the personal belief of the reviewer. And even the argument on the disruptive character of the Cys->Ser mutations when in hydrophobic pockets seems controversial. According to PAM120 replacement matrix, the Cys->Ser replacement is neutral (scoring 0), whereas the Cys->Ala one is unfavorable (scoring -3). Different scorings are assigned, instead, in BLOSUM62, with nearly equivalent estimates for Ala (0) and Ser(-1). At any rate, the three cysteines of Nsp9 are close to the surface and not buried in hydrophobic pockets. Moreover, we wish to point out that our conclusions were based on a detailed comparison of the NMR spectra of wild-type and triSer variants of Nsp9. As discussed in our former publication (Esposito et al., Adv. Biol. 2021), the HSQC maps of wild-type and triSer Nsp9 were indeed observed to differ by the loss, in the mutant spectra, of resonances that match the inter-dimer contacts of the tetrameric structures of SARS-CoV-2 Nsp9 (Zhang et al., Mol. Biomed. 2020) and SARS-CoV Nsp9 (Miknis et al., J. Virol. 2009). The missing signals are indicated in Fig. 2E of our Adv. Biol. 2021 paper with wild-type assignments obtained from BMRB. On the other hand, the patterns of the observable NH correlations in the HSQC maps of wild-type and triSer Nsp9, albeit not identical, were essentially similar and consistent with limited chemical shift deviations plausibly reflecting solvation differences. The intermediate exchange dynamics of dimerization and tetramerization were also proven comparing the diffusion coefficients of the two protein variants and hen egg white lysozyme. After expressing his interpretation on the comparison between wild-type Nsp9

and triSer mutant, with “supporting” considerations on Ser substitution for Cys, Dr. Littler adds that ”most of the peaks lost correspond the changes seen in 8DQU”. From his writing, it is not clear which set of peak loss he refers to. As 8dqu is the structure of the Nsp9 complex with 2NSP23, the concerned peak loss should be the one occurring upon titration of wild-type Nsp9 with the nanobody. This peak loss is described in great detail in S1 Table of Supplementary Information of the manuscript under consideration. As explained at length in the text, that progressive loss of backbone NH resonances maps the different Nsp9 epitope regions as well as the Nsp9 inter-subunit contacts (within the dimer and the tetramer). We never observed changes of chemical shifts upon titration with 2NSP23 and 2NSP90. We only observed progressive signal losses on increasing the titrant nanobody concentration. Such a pattern is not at all compatible with the profound structural change of Nsp9 depicted by 8dqu. If the Nsp9 subunits complexed with 2NSP23 had undergone the transition from the canonical folding to the one observed in 8dqu, a simultaneous change of the vast majority of the chemical shifts should have occurred which, under conditions of intermediate exchange, would have led to simultaneous broadening of all resonances. Therefore the statement of Dr. Littler is against the experimental NMR evidence, even the one he himself published (Pan et al., PlosOne 2023) and misinterpreted by attributing the signal loss to the size of the Nsp9-2NSP23 complex. Paradoxically, Pan and colleagues conclude their NMR analysis stating that their “results are broadly consistent with previously reported titration experiments and confirm a central role of Trp-53 within the epitope [25]”, where reference [25] is Esposito et. al., Adv. Biol. 2021. Why then “the widespread broadening” of the HSQC peaks was not deemed to match the changes seen in 8dqu, whereas such a matching is envisaged only for the peak loss we report?

I cannot comment on 2nsp90 as it’s CDR3 loop is distinct from the nanobody I published upon. I drew my sequences for both antibodies from page 19 of the authors supplementary material ADBI-5-2101113-s001.pdf. If major differences in behaviour are being observed can the authors quickly confirm this sequence is correct.

The sequences on page 19 of Adv. Biol. 5, 2101113, 2021 Supplementary Information are correct and the corresponding nanobodies do not exhibit major behavior difference in the Nsp9 titration experiments (S1 Table of Supplementary Information of the manuscript under consideration).

In my hands 2nsp23 and 2nsp90 certainly bind Nsp9 but direct affinity measurements via SPR and ITC appeared poor for an antibody. Hence the absence of this data in Pan et al. It remains remotely possible these antibodies behave differently when expressed via mammalian systems. I had assumed the poor binding to be due to the requirement of the structural change leading to slower than normal on-rates. It would take significant work to assess whether this is true, I see this as a potentially interesting mechanism by which to inhibit a protein and may aid the inhibitory potential of the LNP system deployed by this group.

We do not have any clue about a different behavior of nanobodies expressed in mammalian systems. The ELISA test of 2NSP23 and 2NSP90 binding to Nsp9 worked satisfactorily in our hands, with nanobodies expressed in *E.coli* WK6. In addition, the two mentioned nanobodies exhibited high expression yields and were therefore selected for further testing. The corresponding data are reported in Tables S1 and S2 of the Supplementary Information in Esposito et al., Adv. Biol. 5, 2101113, 2021. In our opinion, any further comment relating the poor nanobody performance to "the structural change leading to slower than normal on-rates" appears superfluous.

I contacted the authors previously about inclusion on the Pan et al manuscript, they were more inclined to want to include discussion on the tetramer form of Nsp9 than I felt comfortable with. Especially as some in the community now even doubt the physiological relevance of the homodimer. I am confident the structure observed is what was present in the crystals and have discussed this with minimal conclusions. I have retained the line requested about the potential for structural influences upon crystallisation but did not to want to include one about Nsp9-tetramerization in the NMR data as I cannot speak to it. I am yet to be convinced by the tetramer interface.

Dr. Littler's version omits to mention that, besides the crystallographic results, the paper by Pan et al. also reports the NMR experiment of Nsp9 titration with 2NSP23 nanobody he and his colleagues interpreted ignoring the picture emerging from the same experiment we had published two years before (Esposito et al., Adv. Biol. 2021). In addition, Pan and colleagues provided a wrong explanation for the resonance loss they too observed, and cited only the minor feature of the Trp53 presence in Nsp9 epitope to support a picture of converging evidence, in spite of totally diverging contexts of interpretation.

The authors are convinced by their proposed mechanism. I don't have any experience with PENELOP interpretation. With enough funds and interest, it would be trivial to obtain new crystal structures from a different condition of Nsp9:2Nsp90 or Nsp9:2Nsp23. Via an independent group if necessary.

I had hoped one of these antibodies would be useful to pull-down Nsp9 within cells or to aid crystallisation for drug screens. Their slow on-rate for wild-type Nsp9 and enforced structural change upon binding limit their use as such tools. If ture, this is useful information for the community. My understanding of certain techniques, and lack of understanding in PENELOP, will naturally bias my conclusions so I will leave them to stand as written. I will happily reinterpret the data however if I or others find clearer structural data to the contrary that is easily interpretable.

We appreciate the availability of Dr. Littler to reconsider his interpretation in the presence of contrary and easily interpretable evidence. We also thank him for kindly leaving "as written" the PENELOP conclusions he does not understand.

Dr. Littler’s poor understanding of PENELOP cannot however condition the acceptance of our manuscript, especially if other reviewers, presumably more competent in that area, had already commented as follows:
i)“The PENELOP technique itself seems sound (has been published by the same group - no citations so far though - in well known and reputable journals (Analytical Chemistry ref 18 and PCCP ref 19) and the analysis of the technique in ref 19 in particular is good.”
ii) “Overall, the experimental execution is solid, and the conclusions supported by the data”.
Nonetheless, Dr. Littler asks for further experimental verifications through new crystallographic determinations ”Via an independent group if necessary”.
In our opinion this request is particularly excessive in the presence of compelling evidence demonstrating i) the impossibility of interpreting the NMR titration data of Nsp9 solutions with both 2NSP23 and 2NSP90 nanobodies in terms of the extensive folding modification observed in the Nsp9-2NSP23 crystal structure reported by Pan and colleagues (8dqu); ii) the successful inhibition of the viral replication in SARS-CoV-2 infected cells due to the nanobody 2NSP23 which was designed to target Nsp9, i.e. an essential component of the viral replication and transcription complex.
We do not intend to cast any doubt on the Nsp9-2NSP23 complex structure obtained by Dr. Littler and colleagues, but sometimes, especially for small proteins, crystal structures faithfully report only the occurrence of artifacts enforced by crystallization conditions.
